# Transplantation of rat-derived microglial cells promotes functional recovery in a rat model of spinal cord injury

**DOI:** 10.1590/1414-431X20187076

**Published:** 2018-07-30

**Authors:** Dewei Kou, Tianmi Li, Hong Liu, Chuansheng Liu, Yanwei Yin, Xing Wu, Tengbo Yu

**Affiliations:** 1 Department of Pain, the Affiliated Hospital of Qingdao University, Qingdao, China; 2 Operating Room, the Affiliated Hospital of Qingdao University, Qingdao, China; 3 Department of Sports Medicine, the Affiliated Hospital of Qingdao University, Qingdao, China

**Keywords:** Microglia, Spinal cord injury, Transplantation, CD68, OX42

## Abstract

This study evaluated the effect of microglia transplantation on neurological functional recovery in rats subjected to traumatic spinal cord injury (SCI). The rat model of SCI was established using a weight drop device. Forty SCI rats were randomly divided into the microglia group and the saline group. Then, rat-derived microglial cells or normal saline was injected into the injured site 7 days after surgery. The Basso-Beattie-Bresnahan (BBB) score, inclined plate test, and motor-evoked potentials (MEPs) were applied to assess the recovery of motor function. Hematoxylin and eosin (H&E) staining was used to assess the therapeutic effect. Microglia transplantation significantly improved BBB scores and functional scores at 2, 3, 4, 6, and 8 weeks after surgery compared to saline injection (P<0.05). Meanwhile, a prolonged MEP latency and decreased MEP amplitude were observed at 4 and 8 weeks in the microglia group (P<0.05). Histological analysis showed less damage and better prognosis in SCI rats of the microglia group. BrdU^+^ cell tracing experiments showed that microglia were recruited to the injured area of the spinal cord at 7 and 14 days after transplantation. The intensity of immunofluorescence was increased in CD68^+^ and OX42^+^ microglia at 2 days, 1 week, and 2 weeks, and then decreased at 3 and 4 weeks after transplantation in the microglia group. The transplantation of activated microglia played a key role in promoting the recovery of spinal cord function in a rat model of SCI.

## Introduction

Spinal cord injury (SCI) is a serious clinical problem. An injury to the spinal cord disrupts the conduction of motor and sensory information between the brain and body (1,2). According to epidemiological surveys conducted over the past 2 decades, the incidence of SCI ranges from 12.7 to 29.7 per million people in developing countries. An increasing number of patients worldwide have endured permanent disability and multiple dysfunctions, including disrupted bowel and bladder, motor, and autonomic functions along with secondary conditions such as intense pain. SCI directly impacts the patient's quality of life (3,4).

The pathophysiology of SCI involves primary and secondary mechanisms of injury. The primary injury is typically restricted to immediate physical injury to the spinal cord due to the laceration, contusion, compression, or contraction of the neural tissue. Pathological changes associated with the primary injury include severed axons, direct mechanical damage to cells, and ruptured blood vessels. The secondary injury is characterized by further destruction of neuronal and glial cells, mainly due to inflammatory responses, leading to the expansion of the injury site and loss of neurologic function. For example, oxidative damage appears and a glial scar is formed (1,5). Although basic science, preclinical, and clinical studies have aimed to block the progressive pathogenesis of SCI and promote neuroregeneration, no proven therapeutic modality exists. Therefore, studies exploring therapeutic strategies for patients with SCI are needed to improve neurological outcomes.

Microglia are derived from mesodermal cells belonging to the hematopoietic lineage and are the macrophage-like cells of the central nervous system (CNS) that are distinguished from other glial cells, such as astrocytes and oligodendrocytes (6). Neuron and glial cells interact to contribute to spinal nerve function and neuronal architecture (7). The microglia in the normal brain are the so-called “resting” or “quiescent” microglia. These cells display a ramified morphology with small somata and long, branched processes (8). Activated microglia or macrophages adopt an ‘amoeboid' phenotype and produce inflammatory mediators (such as IL-beta, TNF-alpha) in response to infectious and traumatic stimuli. Microglia modulate both the activation and down-regulation of an immune response in the CNS (6,9). In addition, microglia secrete neurotrophic factors to support neural cells. Strategies that regulate microglial function may be useful for the prevention of neurological dysfunction in a variety of CNS injuries or neurodegenerative diseases (10). As shown in the study by Narantuya et al. (11) microglia transplantation attenuates white matter injury in a rat model of chronic ischemia. Mukaino et al. (12) found that an anti-IL-6-receptor antibody promoted the repair of spinal cord injury by inducing microglia-dependent inflammation. According to Takata et al. (13) microglia transplantation exerts a therapeutic effect on a rat model of Alzheimer's disease. These studies have provided encouraging results regarding the effectiveness of microglia transplantation in treating SCI.

Currently, cell transplantation therapies have become promising strategies in pre-clinical research on SCI (14). The common transplanted cell types include Schwann cells, olfactory ensheathing glial cells, neural stem/progenitor cells (adult and embryonic), and mesenchymal stem cells (14–17). However, very few reports on microglia transplantation in SCI are available. We aimed to explore the therapeutic potential of rat-derived microglia transplantation into a rat model of traumatic SCI.

## Material and Methods

### Isolation and identification of primary rat microglia

#### Isolation

Postnatal 24-h specific pathogen-free (SPF) Wistar rats were provided by the Drug Control Center of Qingdao City (China). All animal experiments were approved by the Affiliated Hospital of Qingdao University Ethics Committee and the National Institutes of Health Guide for the Care and Use of Laboratory Animals (No. 2015028).

Primary microglial cells were prepared from brain hemispheres, mainly the corpus callosum, which were dissected from anesthetized suckling mice under sterile surgical conditions. After washes with PBS, the brain hemispheres and corpus callosum were dissociated and digested with trypsin for 60 min. Then, the undigested tissue was removed by filtering through 200 mesh sieves. The filtrate was transferred to a centrifuge tube, and complete DMEM/F12 (Sigma-Aldrich, China) medium (including 10% FCS (Gibco), 100 U/mL penicillin, and 100 μg/mL streptomycin) was added to terminate the digestion. The supernatant was discarded after centrifugation at 4 *g* for 5 min at 37°C. Cells were resuspended in complete DMEM/F12 medium and maintained in T 75 cm^2^ flasks in a 37°C incubator with a 5% CO_2_ atmosphere. After 5 days of primary culture, microglial cells were harvested from mixed glial cells by mechanical oscillations induced by shaking the flasks on a rotary shaker at 260 rpm/min for 2 h or by incubating the cultures with 0.125% trypsin (Shanghai Ruji Biotechnology Development Co., Ltd., China) for digesting before shaking. Then, 12 mmol/L lidocaine hydrochloride (pH: 7.2–7.4) was administered 10 min during culture to activate the microglia.

#### Identification

Microglial cells were identified by immunocytochemical staining using the ABC method. Cells of the 3rd generation growing on a 6-well plate were fixed with alcohol and acetone (1:1). Cells were blocked with a 3% BSA/PBS solution for 30 min, incubated with a rabbit anti-CD68 antibody (bs-0649R, BIOS, 1:200; Shanghai Jianglai Biotechnology Co, Ltd., China) and a mouse anti-OX42 antibody (550299, BD Pharmingen^TM^ Technical, 1:50; Shanghai Jianglai Biotechnology Co, Ltd.) for 1 h, washed with PBS, and then incubated with anti-rabbit IgG-Cy2 (C2306, Sigma, 1:400) and anti-mouse IgG-Cy3 (078–18-061, KPL, 1:400) antibodies for another 1 h. PBS was added to cells in the control group instead of the anti-CD68 and anti-OX42 antibodies.

### Proliferation and attachment of primary rat microglia

#### Proliferation

The 1st, 2nd, 4th, 6th, and 8th generation of microglia cells were seeded in 24-well plates at a density of 1.0×10^4^ cells/mL. Cells in 3 wells were counted from 1 to 9 d to assess cell survival. The growth curve was constructed with time (d) as the abscissa and the number of cells as the ordinate.

#### Attachment

The 2nd, 4th, 6th, and 8th generation of microglia cells were digested and seeded on 24-well plates. Cells in 4 wells were counted at 2, 4, 6, 8, and 10 h after seeding. A cell attachment curve was constructed according to the cell attachment rate, which was calculated as follows: cell attachment rate (%) = adherent cell count / seeded cell count ×100.

### Rat model of SCI

#### Animal and study design

Twenty adult female Wistar rats (0.19–0.22 kg) were randomly assigned to two groups: the experimental group A (n=10) that received SCI surgery and received the microglial cell transplantation, and the control group B (n=10) that underwent the sham surgery and received a saline injection.

#### SCI model

A modification of Allen's weight-drop technique was used to create moderate SCI. Rats were anesthetized by intraperitoneal administration of 10% chloral hydrate (1.5 mL/kg) before surgery and fixed on the operating table in the prone position. Surgical sites were determined by locating the 13th thoracic vertebra of floating ribs. After shaving, the animal's skin was sterilized with an iodine tincture and medicinal alcohol and then covered with a sterile sheet. A 3-cm incision was made on the median dorsum extending into skin and subcutaneous fascia; then paravertebral muscles underwent blunt dissection from both sides. The T7-T9 spinous process was exposed, the T8 spinous process was excised, and the T8 lamina was removed to expose spinal epidural. A 6 g cylindrical weight was dropped from a 10 cm height through a hollow glass tube (2 mm diameter) onto the spinal cord. The following criteria were used to assess the successful establishment of the model: rats presented bilateral hindlimb twitching and tail flicking, followed by complete relaxation and mopping on the ground after awakening from anesthesia. Subsequently, the muscle and incision were sutured layer-by-layer and closed. Analgesia and infection prevention treatments were administered after the operation via an intraperitoneal injection of ketamine (10 mg/kg) and an intramuscular injection of gentamicin (5 mg/kg).

### Transplantation of primary rat microglia

#### Microglial cell transplantation

At 7 days after SCI surgery, the initial incision was exposed to allow the injection of 2×10^6^ microglia cells in the center of injured spinal cord of group A using a 10 µL micro-syringe with 75° angle. A gelatin sponge tamponade was placed in the hole of the spinal cord to prevent cell loss after the syringe was withdrawn. Meanwhile, 0.9% normal saline was injected into rats in group B at a similar site.

### Evaluation of rat motor function

#### Basso-Beattie-Bresnahan (BBB) score

Functional outcomes were measured using the BBB score as described by Gale et al. (18). All behavioral tests were conducted by two “blinded” investigators at 1 day (d), w), 2 w, 3 w, 4 w, 6 w, and 8 w after surgery. Scores ranged from 0–21 points, where 0 indicated complete paralysis and 21 indicated normal function, and were used to assess hindlimb movement, joint movement, gait stability, and the position of the claw and tail. Observations were performed at 7–11 pm daily, and 4–5 min were required to observe each rat along with bladder emptying.

#### Inclined plate test

The inclined plate test was performed as described by Rivlin et al. after BBB scores were recorded 19. Rats were placed on a smooth wood plate covered with a rubber pad and the inclined plate could rotate around its bottom. The body axis was parallel to the vertical axis of the plate. The plate angle increased by 5° in each trial. The maximum angle at which the rat maintained its position for 5 s was considered its functional score. Each rat was measured 5 times and the mean value was recorded as the final score.

#### Motor-evoked potentials (MEPs)

MEPs were measured in all rats in groups A and B immediately after surgery and at 1, 4, and 8 w after the operation. A normal rat was tested as a reference. The sterile dorsum skin was incised and exposed. The stimulating electrode was slowly inserted into the intervertebral space between T5 and T6. The reference electrode was inserted into the paraspinal muscle at the same level. Two recording electrodes were inserted into gastrocnemius muscle, with a 1-cm interval between positive and negative polarity. The ground wire was inserted into the rat’s abdomen at a penetration depth of 5 mm. The stimulus parameters were a frequency of 1 Hz, a duration of 0.2 ms, and an intensity of 5 mA. T5–T6 spinal cord was stimulated with a single pulse wave.

### Histological and immunofluorescence staining

#### Study design

Twenty adult female Wistar rats with SCI were randomly divided into two the following two groups: 1) Group A: activated microglial cell transplantation group, n=10; 2) Group B: a sham-operated control group that received a normal saline injection, n=10. At 2 d, 1 w, 2 w, 3 w, and 4 w after surgery, rats were sacrificed and injured spinal cords were harvested. Samples were fixed with 4% paraformaldehyde overnight and embedded in paraffin or stored in liquid nitrogen for frozen sections.

#### Bromodeoxyuridine (BrdU) tracing

Rats were injected with 5 μL of the BrdU antibody and 2×10^6^ microglial cells (mixed in H-DMEM and 10% FBS for 12 h) at 7 d after SCI surgery for the histopathological analysis of proliferating cells at the lesion sites.

#### Immunofluorescence staining

Animals were re-anesthetized and transcardially perfused with normal saline followed by 4% paraformaldehyde in 0.1 mol/L phosphate-buffered saline (PBS). The spinal cord was removed and immersed in the same fixative at 4°C for 24 h. A spinal segment centered over the lesion epicenter was transferred into a 20% sucrose solution in PBS at 4°C overnight and embedded in optimal cutting temperature compound. The embedded tissue was immediately frozen in liquid nitrogen and stored at –20°C until further use. Frozen sections were cut on a cryostat and incubated with the following primary antibodies overnight at 4°C: rabbit anti-CD68 antibody (bs-0649R, BIOS, 1:200), mouse anti-OX42 antibody (550299, BD Pharmingen^TM^ Technical, 1:50) and anti-BrdU antibody. Sections were then incubated with secondary antibodies for 1 h at 37°C. Negative control experiments included a normal spinal cord section incubated with primary and secondary antibodies. Fluorescence was visualized on Leica DMI4000B (Germany) inverted microscope, and 5 fields were examined at 400× magnification under the same exposure conditions. A computer-assisted image analysis Leica-Qwin-V3.3.1 system was used to count the number of microglial cells. Three observers who were blinded to this study protocols analyzed the images.

#### Hematoxylin and eosin (H&E) staining

Paraffin sections were generated and stained with H&E. Two independent blinded observers examined fields in each sample.

### Statistical analysis

Statistical analysis was performed using SPSS 11.5 software (SPSS, USA). All data are reported as means± SD. Student’s *t*-test was used to analyze between-group differences. One-way ANOVA was used to detect multivariate differences. A P value <0.05 was considered statistically significant.

## Results

### Rat microglial cell identification, proliferation, and attachment

**Figure 1.**
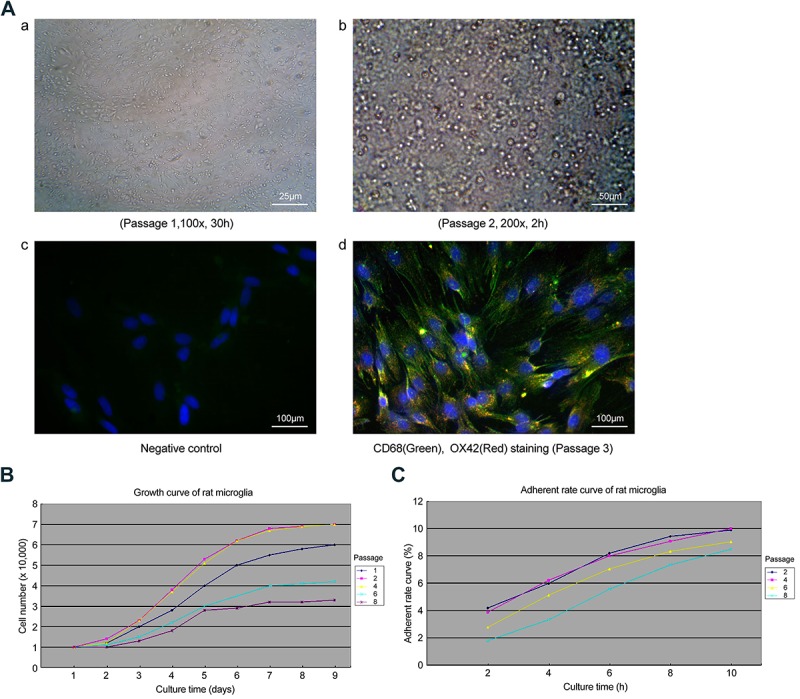
*A*. Morphology of microglia observed under a microscope (*a*,*b*; bars: 25 μm and 50 μm, respectively) and images of immunocytochemical staining for microglia stained with CD68 (green), OX42 (red) and a nuclear marker (blue) (200×) in microglia (*c*,*d*; bars: 100 μm). *B*, Growth curve of different passages. *C*, Adherent rate curve of different passages.

Rat microglial cells were isolated from the early postnatal SPF Wistar rat brain, and images of the 1st and 2nd generation of cultured cells are shown in Figure 1A (a,b). Microglial cells exhibited a rounder shape and a strong refractive index, with some floating cells and cells displaying an irregular edge. The immunocytochemical staining showed positive staining for CD68 (green) and OX42 (red), specific cell-markers for microglia, in the 3rd generation of cultured cells, as shown in Figure 1A (c,d). According to the cell counting analysis, the 1st, 2nd, and 4th generations of microglial cells displayed stronger proliferation than did the 6th and 8th generations of cells, and rapid growth was observed from approximately the 3rd–6th day (Figure 1B). The attachment rate was approximately 90% for the 2nd and 4th generation cells within eight hours, and approximately 80% for the 6th and 8th generation cells (Figure 1C).

### Hindlimb motor function

**Figure 2.**
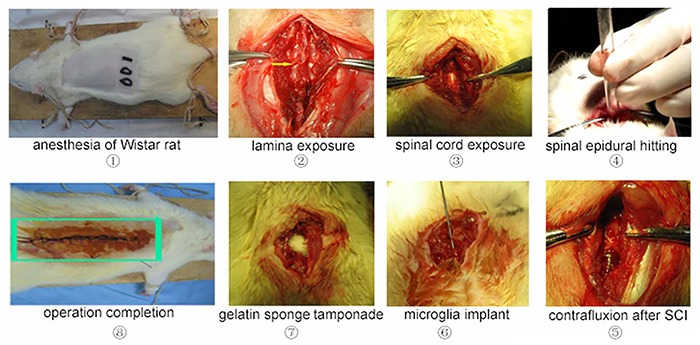
Process used to establish the rat model of spinal cord injury.

**Figure 3.**
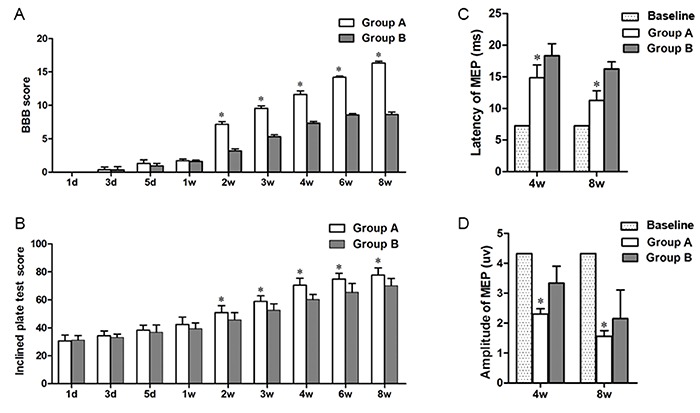
Evaluation of the functional recovery of the spinal cord in spinal cord injury rats between microglial cell transplantation group A and saline injection group B. *A*, Basso-Beattie-Bresnahan (BBB) score; *B*, Inclined plate test score; *C*, Latency of motor-evoked potentials (MEP); *D*, Amplitude of MEP. Data are reported as means±SD. *P<0.05 group A compared to group B (*t*-test). d: day(s); w: week(s).

The SCI model was established as shown in Figure 2. Motor function score analysis revealed significantly higher hindlimb BBB scores for group A than group B at 2, 3, 4, 6, and 8 w after surgery (P<0.05), whereas a significant difference was not observed at 3 d, 5 d, and 1 w after surgery (P>0.05). All rats presented the behavior of bilateral hindlimb mopping on the ground at 1 d, with BBB score of 0 (Figure 3A). Based on the results of the inclined plate test, the amp angle of the test was significantly higher in group A than that in group B at 2, 3, 4, 6, and 8 w after surgery (P<0.05); the difference was not significant at 1 d, 3 d, 5 d, and 1 w (P>0.05) (Figure 3B), consistent with the BBB scores. After stimulation of the spinal cord, stable normal waveforms of MEPs were recorded in the normal rats (latency: 7.256 ms, peak value: 4.326 uv). However, waveforms were not recorded immediately after surgery or at 1 w after injury in groups A and B. A prolonged latency and a decrease in the amplitude of MEPs were observed in both groups A and B. Greater improvements were observed at 8 w after injury than at 4 w after injury. Significantly greater MEP latency and amplitude were observed in group A than group B.

### Activated microglial cell transplantation

**Figure 4.**
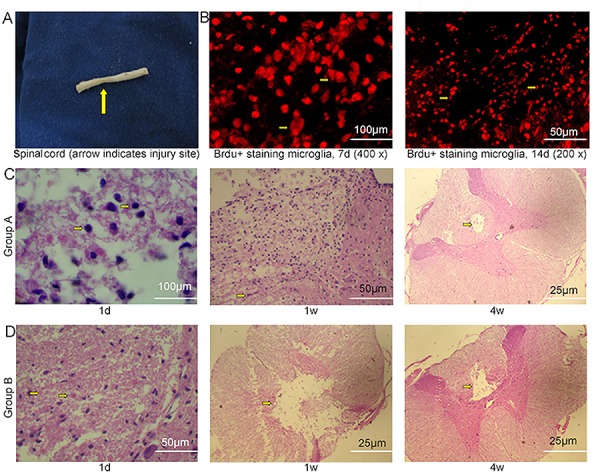
Tracing of transplanted microglial cells and histological staining of the injured spinal cord. *A*, The injured spinal cord. *B*, BrdU-labelled microglia after transplantation (7d, 400×, bar: 100 μm; 14d, 200×, 50 μm). *C* and *D*, H&E staining of the injured spinal cord in the microglial cell transplantation group A and saline injection group B at 1 day (d), and 1 and 4 weeks (w). Bars: *C*: 100 μm, 50 μm, 25 μm, respectively; *D*: 50 μm, 25 μm, 25 μm, respectively.

The microglial cell transplantation in SCI rats using the micro-syringe method was simple and feasible. The injured spinal cord was obtained and images are displayed in Figure 4A. BrdU^+^ cells were used to trace transplanted microglial cells. The transplanted microglia accumulated in the injured spinal cord at 7 d after transplantation and survived very well at 14 d (Figure 4B). H&E straining in group A showed a patchy distribution of microglial cells (with a similar round shape) around the transplanted site at 1 d. Microglial cells (with an amoeba or columnar shape) were present in a patchy distribution around liquefaction necrotic foci (with glial fibers similarly located around the site) at 1 w. However, over time, the number of microglial cells was gradually reduced. Most of the liquefaction necrotic foci disappeared and a polycystic cavity existed in a small part of the area at which microglial cells had accumulated around at 4w (Figure 4C). H&E straining in group B showed a patchy distribution of microglial cells (mostly the amoeba or columnar shape) and blood vessel congestion in the spinal cord grey matter at 1d after saline injection. Scattered liquefaction necrotic foci appeared in the grey and white matter at 1 w, which were surrounded by microglial cells and glial fibers moving around. Over time, the area of liquefaction necrotic foci and the number of microglial cells decreased. A small number of microglial cells accumulated around liquefaction necrotic foci at 4 w (Figure 4D). The liquefaction necrotic foci disappeared earlier and the size of polycystic cavity decreased in group A compared with group B at the end of the observation period.

**Figure 5.**
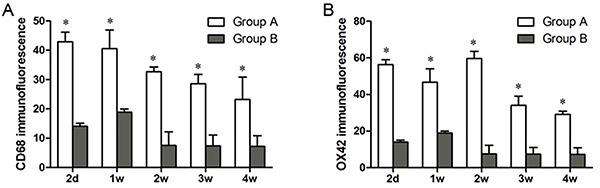
Immunofluorescence staining of microglial cells in the microglial cell transplantation group A and saline injection group B. *A*, CD68^+^ immunofluorescence intensity; *B*, OX42^+^ immunofluorescence intensity. Data are reported as means±SD. *P<0.05 compared to group B (*t*-test). d: day(s); w: week(s).

CD68- and OX42-labeled microglial cells exhibited higher intensity immunofluorescence staining (activity) at 2 d, 1 w, and 2 w, which decreased at 3 w and 4 w. Importantly, both CD68^+^ and OX42^+^ immunofluorescence intensities were significantly higher in group A than in group B (Figure 5A and B).

## Discussion

Treatments that prevent or reverse SCI are not available worldwide; thus, various rehabilitative cellular and molecular therapies have been tested in animal models (20). To identify whether the microglia transplantation method was unambiguously effective, we assessed the neurological functional recovery in rats after traumatic SCI and found that the recovery of hindlimb motor function in SCI rats treated with microglia transplantation was significantly increased compared with that in control group. Furthermore, the injured area was reduced after microglia transplantation.

Microglia were initially derived from neonatal brains typically using proteolytic or mechanical means of dissociating cells from CNS tissues, as previously reported (4,21). Cultured microglial cells are usually isolated from mixed glial cultures after these mixed cultures have been maintained for 10 days or longer *in vitro* (22). Here, we have made some improvements to the classic method for separating microglia, such as repeated rinses and the removal of blood vessels during operation to avoid mixed macrophages. Culture bottles were also pretreated with poly-lysine to promote the rapid adhesion and proliferation of microglia, and we increased the density of inoculated cells and the frequency of culture media exchange to reduce the culture time. Microglial cells were harvested after 5 d of primary culture, and then immunocytochemical staining was performed using OX42 and CD68 antibodies. CD68 and OX42 antibodies (which recognizes the CD11b antigen) form immunoreactive products that enable the visualization of microglia (7). The monoclonal OX42 antibody against CD11b recognizes both activated and resting microglia, and rat CD68 has been used as a marker of fully activated microglia in brain lesions (8,23). The 3rd generation of cultured cells was positively stained for CD68 (green) and OX42 (red), suggesting that we successfully isolated microglia from rats.

The traumatic SCI models described in the literature employed blunt contusion, compression injuries, or sharp injuries (14); the weight drop method was adopted in this study. Usually, microglia are directly transplanted into the injury site or adjacent to it by injecting a few microliters of a cell suspension (with several hundred thousand cells) with fine needles at 1–2 weeks after the injury (14). In our study, microglia were micro-injected into the injured spinal cord along the previous incision at 7 d after surgery. Rats treated with normal saline served as controls.

Behavioral tests were performed to assess the motor function of SCI rats after microglia transplantation. The American Spinal Injury Association has proposed international standards for the neurological classification of SCI and has generated scores to characterize sensory/motor function and determine the completeness of the injury (3). We compared the BBB scores and the functional scores on inclined plate test between the two groups. The BBB score is a sensitive and reliable method and has been used to predict functional recovery (24). Statistically significant differences were found between the microglia transplantation group and saline injection group from 2 w to 8 w after surgery. Therefore, microglia transplantation exerted a positive effect on the recovery of motor functions. Consistent with these findings, the combination of propofol injection and BMSC transplantation improved hindlimb motor function in rats with SCI, as evidenced by higher BBB and inclined plate test scores (25). Meanwhile, Tan et al. (26) reported that a local injection of Lenti-Olig2 at the lesion site improved axonal conduction in rats with SCI, as determined by measuring MEPs. In the present study, electrophysiological measurements were performed. MEPs were recorded from the injured spinal cord to detect spinal cord-evoked potentials, and the latency reflected the speed of nerve impulses (24). The latency was prolonged and the amplitude was decreased at 4 w and 8 w after transplantation. Significantly greater changes in MEP latency and amplitude were observed in the group that received the microglia transplantation compared with the group treated with saline.

In addition to improving the motor function, microglia transplantation has also been shown to promote the functional recovery of the injured spinal cord. Transplanted cells labelled with BrdU were present in the injured area of the spinal cord at least 7 and 14 d after transplantation, suggesting that the transplanted microglia survived and proliferated to some amount. Consistent with the findings from a previous study suggesting that the microglia displaying an amoeba or columnar shape represent fully activated cells (27), the H&E staining showed that the microglia were activated in this study. Activated microglia release a large number of cytokines to modulate the immune response. According to Hynds et al., the microglia transplantation after SCI promotes the regeneration of sensory nerve fibers (28). In the study by Ogawa et al. (29), transplantation of *in vitro* expanded fetal neural progenitor cells resulted in neurogenesis and functional recovery in adult rats with a spinal cord contusion injury. Moreover, transplanted donor progenitor cells underwent neuronal differentiation within the adult rat spinal cord 5 weeks after transplantation, according to the histological analysis. Similarly, in the present study, the histological results showed that the transplanted microglia survived at injury sites in the spinal cord for a long period after transplantation, reducing the cavity area at 4 w after transplantation.

The OX42 antigen is rarely expressed in resting or dormant microglia and the OX42 antibody generally labels activated microglia. In the present study, the fluorescence intensity of CD68^+^ and OX42^+^ cells was maintained at a relatively higher level at 2 d, 1 w, and 2 w, and then decreased at 3 w and 4 w after transplantation, as determined by immunocytochemical staining. Therefore, we speculated that the activated microglia cells exerted a positive effect on the early phase of injury recovery and gradually returned to the resting state to avoid the release of toxic material by excessively activated cells that damages neurons.

In conclusion, when transplanted into the spinal cord at 7 d after weight drop injury of the spinal cord, rat-derived activated microglial cells migrated to the injured area, improving functional recovery of SCI rats. Thus, microglial cells might be a promising cell source for SCI treatment.
